# Suprapubic catheter insertion: a multicentre review on mortality during short-, medium- and long-term follow-up

**DOI:** 10.1308/rcsann.2025.0058

**Published:** 2025-07-24

**Authors:** C Rao, M Taheem, I Nnorom, S Veer, T Mahesan, N Faure Walker, T Nitkunan

**Affiliations:** ^1^ Epsom and St Helier University Hospitals NHS Trust, UK; ^2^ King’s College Hospital NHS Foundation Trust, UK; ^3^ Surrey and Sussex Healthcare NHS Trust, UK

**Keywords:** LUTS, Catheters, Suprapubic catheter insertion, Frailty, Ageing

## Abstract

**Introduction:**

The aim of this study was to improve shared and informed decision making regarding real-world mortality rates caused by suprapubic catheter (SPC) insertion.

**Methods:**

We undertook tri-centric, retrospective data collection from all patients identified as having a SPC inserted between 2017 and 2022. Data including demographics, frailty scores, comorbidities, reason for SPC insertion and mortality information were collected and statistically analysed.

**Results:**

Sixty-eight patients were identified and included in this study. Mortality after SPC insertion at 30 days was 1.5%: 18.8% at 1 year and 30.4% at 2 years. Medium-term mortality rates were 45.2% at 3 years, rising to 54.5% at 4 years. Patients aged 71 or older at the time of SPC insertion had an overall mortality of 45.9% compared with 10.3% in those aged under 71 years (*p* < 0.01). Mortality was 53% in frail patients who underwent SPC insertion (Rockwood Clinical Frailty Score >3). Limits of the study include the small sample size and heterogeneity of the population.

**Conclusions:**

SPC insertion is associated with a significant mortality rate of 18.8% at 1 year rising to 45.2% at 3 years in our study cohort. Patients with increasing age and frailty may also be at a greater risk of mortality after SPC insertion. This information is in keeping with the limited data available in the published literature and should be highlighted to clinicians and patients when considering SPC insertion.

## Introduction

It has been estimated that there are more than 90,000 people using long-term indwelling catheters in the United Kingdom (UK) and 75% of these patients are aged over 70.^1^ As per the Chief Medical Officer’s Report in 2023, those aged 85 and older will comprise 7% of the UK population by 2066 and therefore adapting home environments to aid independence is vital to improve future practice.^2^ An indwelling catheter can be urethral or placed suprapubically. Indwelling suprapubic catheter (SPC) insertion involves placement of a catheter directly into the urinary bladder, superior to the pubic symphysis.^3^ Indications for SPC use include acute urinary retention in the emergency setting if urethral catheterisation is difficult or dangerous, chronic urinary retention and long-term continence management. These catheters have the benefit of being less traumatic to the urethra, more comfortable and easier to change. Although a key urological technique, there are limited data on outcomes in patients who undergo SPC insertion. Some studies have seen modest reductions in infection rates with SPCs when compared with indwelling urethral catheters Another study found a 30-day mortality rate of 1.8% in those with SPC insertion, associated with intraoperative bowel injury and comorbidities in the perioperative period.^4–6^ A review of current literature revealed only one study with medium- to long-term follow-up specifically in patients with spinal cord injury.^7^ In this study of 1,000 consecutive SPC insertions, mortality was 21.1% (*n* = 182) during the follow-up period of a median of 3.3 years (4 weeks to 16.45 years).

The National Health Service (NHS) Model Health System allows for data-driven quality improvement and providers are encouraged to view their operative procedures and compare performance with other hospitals, regions and peers. This demonstrates a national 30-day mortality rate after SPC insertion of 4.8% and 16.9% within 1 year (Model Health System, accessed June 2024). Frailty is also associated with increased mortality postoperatively and can increase the severity of symptoms in minor urological operations.^8–10^

In this study, we aimed to establish the short-, medium- and long-term mortality associated with SPC insertion and to identify factors that may be linked with mortality.

## Methods

### Recruitment

This was a multicentre retrospective study across three urological units in the UK. Patients were identified through data informatics requests on 22 July 2022 at the three institutions using standard operative nomenclature of ‘cystostomy’ and ‘insertion of suprapubic catheter’ and ‘percutaneous suprapubic catheter insertion’.

### Ethics approval

The study was considered to be an audit and no patient-identifiable or organisation-specific data were collected. Further NHS research committee or Health Research Authority permission was therefore not sought.

### Data collection

Patients identified as having a newly inserted SPC between April 2017 and April 2022 were included in the study. Information regarding procedural complications, patient demographics, urine microscopy and culture, blood investigations and mortality was collected using a combination of electronic and paper notes. Furthermore, past medical history from clerking notes was used to identify comorbidities. Welch’s *t*-test was used to test for statistical significance. The Rockwood Clinical Frailty Score (CFS) was used to assess frailty.^11^ This is a validated assessment tool that assesses activities of daily living, age and dependency on carers to provide a numerical frailty score.

## Results

Sixty-eight patients were initially identified across all three sites of whom two were excluded owing to incomplete data sets. The mean age was 68 years (range 27–92) and 70.5% (*n* = 48) of the patients were men (Table 1). Some 77.9% (*n* = 53) were diagnosed with cognitive impairment, documented as either mild cognitive impairment or dementia in their past medical history, and 51.5% (*n* = 35) had a neurological disorder (Table 1). The follow-up period was between 3 months and 5 years. For 79% (*n* = 50) of patients, SPC insertion was permanent with ongoing catheter changes occurring in the community. In those who had SPC removal (*n* = 13), four underwent surgical procedures (urethral stricture surgery, *n* = 2; bladder outflow obstruction surgery, *n* = 2) and the remainder were managed with other bladder-drainage options including intermittent self-catheterisation and long-term urethral catheterisation. The permanence of SPC insertion was not recorded in the remainder (*n* = 5).

**Table 1:** Patient demographics

**Age**	
Range	27–92
Mean	68
**Gender [*n* (%)]**	
Male	48 (70.5)
**Comorbidities [*n* (%)]**	
Type 2 diabetes mellitus	10 (14.7)
Recurrent urinary tract infection	13 (19.4)
Neurological disorder	35 (51.5)
Cognitive impairment	53 (77.9)
Ischaemic heart disease	27 (39.7)
Previous abdominal surgery	13 (20.6)
History of urological malignancy	2 (2.9)
Obesity	6 (8.8)
**Functional status [*n* (%)]**	
Mobility adjunct use	29 (42.6)
Frailty, CFS ≥3 [*n* = 43]^a^	28 (41.2)

^a^Only subjects >65 years old were considered

Mortality at 3 years was 45.2% (19 of 42) rising to 54.5% (6 of 11) at 4 years (Table 2). The medium-term follow-up mortality rates were 18.8% (12 of 64) at 1 year and 30.4% (17 of 56) at 2 years. Early mortality was 1.5% (1 of 68) by 30 days (Table 2).

**Table 2:** Mortality rates after suprapubic catheter insertion

Time	No. of patients	No. of patients deceased (%)
30 days	68	1 (1.5)
90 days	66	3 (4.5)
1 year	64	12 (18.8)
2 years	56	17 (30.4)
3 years	42	19 (45.2)
4 years	11	6 (54.5)
5 years	2	1 (50)

Overall mortality over the 5-year period in patients aged <71 years at the time of SPC insertion was 10.3% (*n* = 3 of 29) compared with 43.6% (*n* = 17 of 39) in those aged 71 or older (*p* < 0.01) (Table 3), when taking into account all of those initially identified. In the short-term, 90-day mortality was 0% (*n* = 0 of 28) in those <71, and 7.9% (*n* = 3 of 38) in those older than 71 (*p* < 0.01). The respective figures were 3.7% (*n* = 1 of 27) and 18.9% (*n* = 7 of 37) at 1 year (*p* < 0.01). At 3 years, those with age ≥71, had a mortality rate of 56.0% (*n* = 14 of 25) compared with 17.6% (*n* = 3 of 17) in those <71 (*p* < 0.01).

**Table 3:** Mortality rate stratified by age

	Mortality rate, *n* (%), stratified by the number of participants followed up for this period*	
Time	Age <71 at time of SPC insertion	Age ≥71 at time of SPC insertion
30 days	0 of 29 (0)	1 of 39 (0)
90-days	0 of 28 (0)	3 of 38 (7.9)
1 year	1 of 27 (3.7)	7 of 37 (18.9)
2 years	3 of 24 (12.5)	8 of 32 (25.0)
3 years	3 of 17 (17.6)	14 of 25 (56.0)
4 years	0 of 4 (0)	6 of 7 (85.7)
5 years	0 of 1 (0)	1 of 1 (100)

SPC = suprapubic catheter. * Participants who suffered mortality during this period, without a specifically recorded date in the data set were not displayed in the table, but used for analysis of overall mortality over the 5-year follow-up period.

Frailty scores were collected for patients aged 65 or older. A frailty score ranging from 4 to 9 indicates levels of frailty, moving from vulnerable to frailty to completely dependent and approaching the end of life, as indicated in Table 4.

**Table 4:** Clinical Frailty Score (adapted from Rockwood *et al*.^11^)

Clinical Frailty Scale Score	Meaning
4	Vulnerable to frailty
5	Mildly frail
6	Moderately frail
7	Severely frail
8	Very severely frail
9	Terminally frail

When data were stratified by CFS (*n* = 43), participants with a CFS ≥4 (*n* = 15) were considered frail. Some 67% (*n* = 10) of patients considered frail also had a verified neurological diagnosis. Overall mortality in frail patients (CFS ≥ 4) who had undergone SPC insertion in this cohort was 53% during the period of follow-up (*n* = 8).

## Discussion

Our mortality following SPC insertion was 1.5% at 30 days, 18.8% at 1 year and 45.2% at 3 years (Table 2).

The 1.5% 30-day mortality rate post-SPC insertion in this cohort aligns with other observational studies with 30-day mortality rates ranging between 0.8% and 1.8%.^6,7,12–14^ To the authors’ knowledge no long-term mortality follow-up data have been reported in the literature for an unselected population who have undergone SPC insertion.

One-year mortality in nursing home residents after SPC insertion was found to be 37.6%. The comparatively higher mortality rate observed by Chou *et al* possibly reflects increased frailty, along with an increased mean age of 80.9. Specifically in the severely frail population, assessed with a claims-based frailty index, 1-year mortality was 44%.^15^ In a similar vein, a case series of SPC insertion in 861 patients with spinal cord injuries^7^ found 59% morbidity and a 21.1% mortality rate over a median follow-up period of 3.3 years (range 4 weeks to 16.45 years). Patients in this study had a median age of 51 years, with an interquartile range of 28. Frailty was not measured; however, comorbidities were graded with American Society of Anesthesiologists (ASA) scores, with 97.9% being ASA grade 2–3.

A 10.3% 5-year mortality rate was observed in patients aged younger than 71 compared with 43.6% in patients aged 71 or over (*p* < 0.01). Although life expectancy is generally considered to be constantly increasing, both age and frailty remain independent risk factors for mortality.^16–19^ The CFS is a quantitative tool used to subjectively measure the frailty level of a patient, based on levels of dependency on others for activities of daily living.^11^ It is scored for 1–9, with 1 being participants who are ‘very fit’ and 9 being those who are terminally unwell. The Acute Frailty Network links CFS to mortality and finds the average all-cause mortality of those with a CFS of 4 after admission to hospital to be 3%, with these numbers increasing to 6% and 11% with a CFS of 6 and 7, respectively.^20^

A North American study looked into all of those in nursing homes undergoing minor urological surgery, including SPC insertion with a sample size of 34,605 and found that 1-year all-cause mortality was 50%, with a 29.3% decline in activities of daily living.^13^ An Icelandic prospective cohort study investigated several indices including cognitive function and mortality after a nursing home admission at 6-monthly intervals.^21^ In the 690-patient cohort, 160 (23.1%) had died within 1 year of admission. Although insufficient evidence exists to define the medium- or long-term mortality rate associated with SPC insertion, evidence shows that patients with comorbidities such as neurological issues, cancers from pelvic organs and urethral stenosis are more likely to suffer from urinary retention that requires SPC insertion.^22^ Our study supports this, with the incidence of cognitive impairment and underlying neurological diagnosis being 77.9% and 51.5%, respectively.

The risks of SPC insertion, the cognitive status of the recipient and frailty, alongside the potential benefits and potential pitfalls of being a long-term SPC user necessitate considered discussion in determining the appropriateness as well as the feasibility of SPC insertion.

### Study limitations

This study had several limitations. The sample size was relatively small, with only 68 participants included in the final analysis. Of these, nine underwent emergency SPC insertion, which is likely to underrepresent the true incidence across the broader population served by the participating centres. This may reflect a local clinical preference against routine emergency SPC insertion when urethral catheterisation is feasible. Participating centres were affiliated with hospitals offering reconstructive urology, regional emergency interventional radiology, spinal cord injury care and major trauma services. Patient identification was based on hospital coding, a process typically carried out by non-clinical staff and therefore susceptible to errors. These potential sources of bias warrant further systematic investigation to determine their impact. In addition, the patient cohort was heterogeneous, with ages ranging from 27 to 92 years, and the data were collected and analysed retrospectively.

The prevalence of obesity, diabetes and ischaemic heart disease was 9%, 14.7% and 37.9% in this cohort. A prospective paired cohort study controlling for variables such as age, frailty and comorbidity would be necessary to isolate the link that SPC insertion has with mortality. Irrespective of this, urologists often pursue SPC insertion in the elderly and frail as a measure to improve quality of life. The question therein lies in whether there is enough evidence to empower patients and their families to make a well-informed decision.

## Conclusion

This study suggests a mortality rate following SPC insertion of 45.2% at 3 years and 54.5% at 4 years. Overall mortality was higher in those aged 71 and older (43.6% (17/39) vs 10.3% (3/29), *p* < 0.01). Although this study does not explore the impact of SPC insertion on quality of life, a significant factor when considering SPC insertion, it serves to better inform clinical decision making while empowering both patients and caregivers with real-world mortality rates.
